# Impact of excessive body fat mass on mortality after lung transplantation

**DOI:** 10.1016/j.xjon.2025.07.009

**Published:** 2025-07-24

**Authors:** Ayako Oshima, Susumu Sato, Yohei Oshima, Daisuke Nakajima, Toyofumi F. Chen-Yoshikawa, Naoya Tanabe, Masaki Ikeda, Yoshito Fujita, Keiko Wada, Hiroshi Date, Daisuke Yabe

**Affiliations:** a Department of Metabolism and Clinical Nutrition, Kyoto University Hospital, Kyoto, Japan; b Department of Respiratory Medicine, Graduate School of Medicine, Kyoto University, Kyoto, Japan; c Department of Respiratory Care and Sleep Control Medicine, Graduate School of Medicine, Kyoto University, Kyoto, Japan; d Rehabilitation Unit, Kyoto University Hospital, Kyoto, Japan; e Department of Thoracic Surgery, Graduate School of Medicine, Kyoto University, Kyoto, Japan; f Department of Thoracic Surgery, Graduate School of Medicine, Nagoya University, Nagoya, Aichi, Japan; g Department of Thoracic Surgery, Japanese Red Cross Wakayama Medical Center, Wakayama, Japan; h Department of Diabetes, Endocrinology and Nutrition, Graduate School of Medicine, Kyoto University, Kyoto, Japan

**Keywords:** lung transplantation, body composition, body fat, nutrition, mortality

## Abstract

**Objective:**

To investigate whether changes in body composition up to 2 years after lung transplantation are associated with long-term prognosis.

**Methods:**

We retrospectively investigated changes in body composition and their association with mortality using a Cox proportional hazards regression model in patients who underwent lung transplantation at Kyoto University between 2013 and 2018. Body components, such as body mass index (BMI), fat-free mass index (FFMI), and body fat mass index (FMI) were obtained at discharge, 6 months, 1 year, and 2 years post-transplantation.

**Results:**

The analysis included 71 patients (39 males, mean age 48.0 years). The BMI, FFMI, and FMI increased significantly until 1 year post-transplantation. The respective distributions of each classification (low, standard, and high) for each body component at 1 year post-transplantation were as follows: BMI, 37.9%, 60.6%, and 1.5%; FFMI, 71.2%, 28.8%, and 0%; and FMI, 33.3%, 36.4%, and 30.3%. The FMI at 1 year post-transplantation was an independent factor, and the group with a high FMI at 1 year post-transplantation had significantly lower survival (hazard ratio, 5.370; 95% confidence interval, 1.396-20.660; *P* = .014).

**Conclusions:**

After lung transplantation, the patients had increased BMI, FFMI, and FMI. The FMI increased above the reference values in an increasing number of patients, and a high FMI at 1 year post-transplantation was associated with a poor prognosis.

## Graphical Abstract

**Figure:**
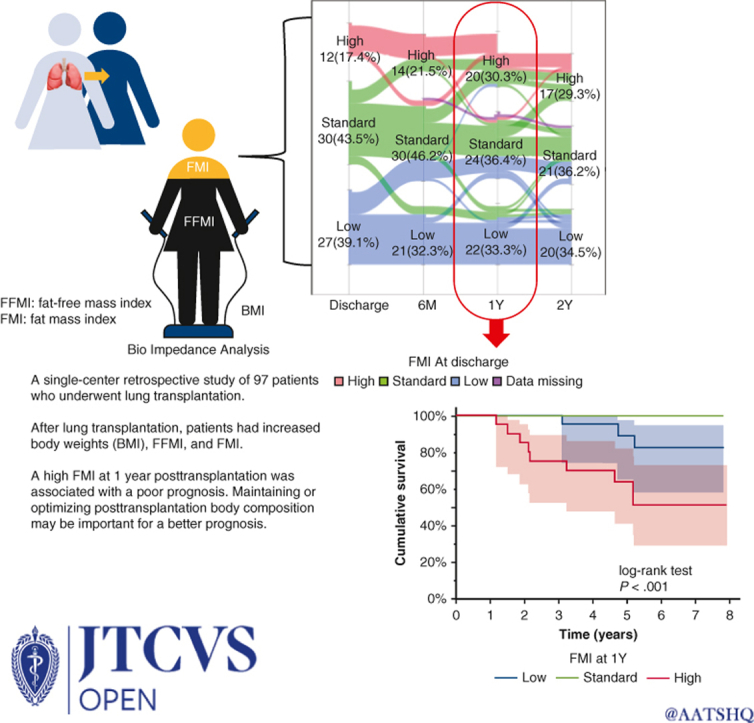

**Figure:**
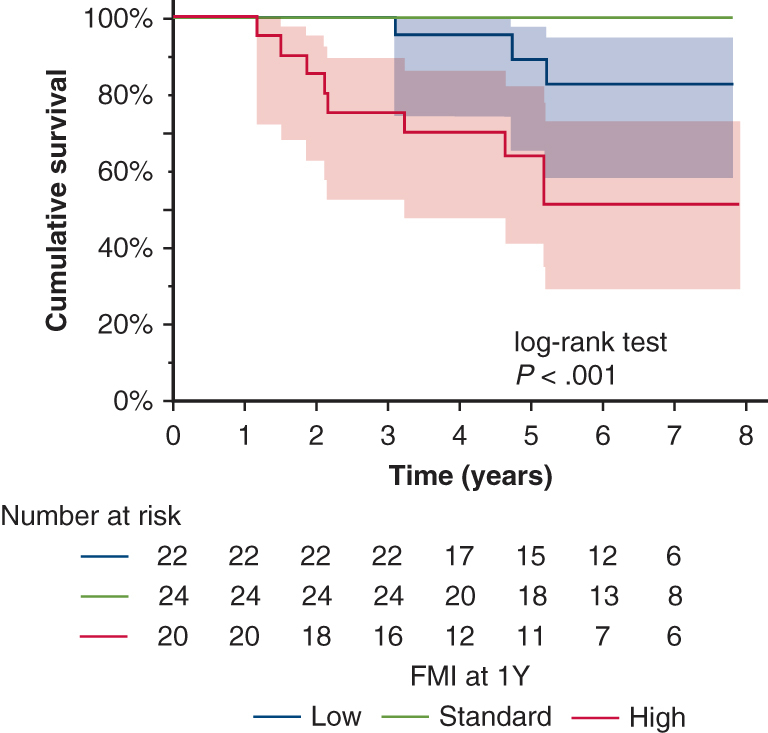
Kaplan‒Meier overall survival curves according to FMI in 1 year post-transplantation.

**Central Message**

After lung transplantation, the fat mass index (FMI) was increased above the reference values in an increasing number of patients, and a high FMI at 1 year post-transplantation was associated with a poor prognosis.

**Perspective**

In this single-center retrospective study of 97 patients who underwent lung transplantation, we found that after lung transplantation, patients had increased body weight, fat-free mass index, and fat mass index (FMI), and that a high FMI at 1 year post-transplantation was associated with a poor prognosis. Maintaining or optimizing post-transplantation body composition may be important for a better prognosis.

Lung transplantation has been performed as a life-saving measure in patients with critical respiratory illnesses. The 5-year survival rate of deceased donor lung transplantation is 73.7% in Japan^1^ and 57.2% in adults according to international registries.^2^ In recent years, peritransplantation nutritional status has been reported to affect the post-transplantation outcomes in lung transplant recipients; poor peritransplantation nutritional status included a pretransplantation status of underweight, sarcopenia, and frailty.^3^, ^4^, ^5^, ^6^, ^7^

Patients with chronic respiratory failure generally have increased basal metabolism due to worsening respiratory status^8^ and elevated inflammatory cytokine levels,^9^ which leads to wasting, anorexia,^10^ and decreased nutritional intake.^11^ Malnutrition and poor prognosis after surgery also have been reported in other organ transplantations and thoracic surgeries.^12^^,^^13^ On the other hand, being overweight or obese also may cause metabolic syndrome and a poor prognosis in patients undergoing lung transplantation.^14^ Several possible mechanisms may underlie the development of overweight/obesity. It has been suggested that chronic respiratory dysfunction itself, which is the primordial cause of underweight, can be ameliorated by lung transplantation.^15^ Systemic steroids administered as immunosuppressants are known to cause obesity through their appetite-increasing and fat-accumulating effects. Consequently, patients tend to gain weight after lung transplantation,^16^ and some may become obese.

In contrast, skeletal muscle mass is likely to decrease during the acute post-transplantation period because of surgical intervention and reduced nutritional intake.^17^ Corticosteroids also can induce muscle atrophy.^18^ Consequently, muscle mass typically does not recover for several years after lung transplantation.^19^

After lung transplantation, body weight and composition are prone to alterations, and an imbalance in body composition is likely to occur, resulting in poor prognosis. However, to date few studies have investigated body composition during the peritransplantation period and for up to 2 years post-transplantation.

There is an urgent need to optimize nutritional management approaches to address weight and body composition after lung transplantation. Considering that the steroid dosage at our institution is maintained after the first postoperative year, we expected that the body composition would change markedly up to the first postoperative year and then plateau. Therefore, we hypothesized that the imbalance in body composition during the first postoperative year would be associated with long-term prognosis after lung transplantation, and that information on both the course and 1 year postoperatively (the breakpoint in the course) would be important for verifying this hypothesis.

## Materials and Methods

### Study Design

In this retrospective cohort study, we investigated changes in body composition and the association between body composition and post-transplantation mortality in patients after lung transplantation. This study was approved by the Ethics Committee of Kyoto University (approval R3678, approved Dec 5, 2022). The need for written informed consent was waived owing to the study's retrospective nature.

### Participants

The study subjects were patients who underwent lung transplantation at Kyoto University between May 1, 2013, and May 31, 2018. The exclusion criteria were patients age <18 years, retransplantation cases, and patients for whom body component analysis was not possible owing to pacemaker placement or lower limb amputation.

### Data Collection

Data on age, sex, underlying lung disease, surgical procedure, steroid medication (including steroid pulse therapy), and insulin administration were collected from medical records.

### Body Composition

Body composition was obtained during nutritional guidance at discharge after transplantation and at periodic medical checkups at 6 months, 1 year, and 2 years post-transplantation. The reason for measuring body composition at 2 years was to ensure that the body composition plateaued after 1 year. Body composition was measured using multifrequency bioelectrical impedance methods (InBody 720; InBody Japan, Inc). Bioelectrical impedance analysis (BIA) was performed for at least 2 hours after fluid and food intake. Generally, the fat-free mass (FFM)—including bone, cardiac muscle, visceral muscle, and skeletal muscle—is calculated by subtracting the fat mass (FM) from the body weight. By using multiple frequencies, this device can calculate the body water, protein, and mineral contents as well as FM. With this device, the FFM is calculated from correlation with the body water content considering individual differences, and the FM is calculated by subtracting the FFM from the body weight. These values were calculated using the manufacturer's software (Lookin’Body 120). Trained healthcare professionals performed all the measurements.

Body mass index (BMI), FFM index (FFMI),^20^ and FM index (FMI)^21^ were calculated as body weight (kg)/height (m^2^), FFM (kg)/height (m^2^), and FM (kg)/height (m^2^), respectively, and classified into 3 groups: low, standard, and high. BMI was defined based on World Health Organization criteria, such as low if 18.4 kg/m^2^ or less, standard if 18.5 to 24.9 kg/m^2^, and high if 25.0 kg/m^2^ or more. Standard FFMI and FMI values were defined according to the study by Kyle and colleagues.^16^^,^^22^ In FFMI, 16.7 to 19.7 kg/m^2^ for men and 14.6 to 16.6 kg/m^2^ for women, and in FMI, 1.8 to 5.1 kg/m^2^ for men and 3.9 to 8.2 kg/m^2^ for women. The FMI differs from body fat percentage, calculated as FM/weight × 100 (%).

Because body composition could not be measured before transplantation, only the pretransplantation BMI was collected from medical records.

### Nutritional Intake and Physical Activity Level

Nutritional intake and physical activity level (PAL) data were obtained using the Food Frequency Questionnaire (FFQ) (FFQg version 2.0, Excel Eiyoukun), which was administered during nutritional guidance at periodic medical checkups at 6 months, 1 year, and 2 years post-transplantation; see the Online Data Supplement for more information. The FFQ was not performed at discharge; therefore, nutritional intake was calculated from the food order history and the nurse's record of intake in the medical records. Moreover, there were no data on PAL at discharge.

### Mortality

The survival status extracted from the database maintained by our hospital's lung transplantation coordinator was used for prognostic analysis. Observations were conducted until October 31, 2021, with termination at the end of the observation period or date of death, whichever occurred first. Post-transplantation body composition was the independent variable. Age, sex, primary disease, steroid medication dose, the presence or absence of steroid pulse therapy, and the presence or absence of insulin administration were used as potentially relevant covariates.

### Statistical Analysis

Data are expressed as mean ± standard deviation or as median and interquartile range (IQR), and categorical data are expressed as frequency. Data changes at discharge and at 6 months, 1 year, and 2 years post-transplantation were compared using repeated-measures analysis of variance, the Friedman test, and the χ^2^ test. A Cox proportional hazards regression model was used to analyze the association between body composition and mortality. Body components and potentially relevant covariates that were significant according to the log-rank test were used in the analysis. Kaplan-Meier survival curves were used to plot survival curves for patients with low, standard, and high values for each body component. All analyses were conducted using SPSS version 27 (IBM). Statistical significance was set at *P* < .05.

## Results

**Figure E1:**
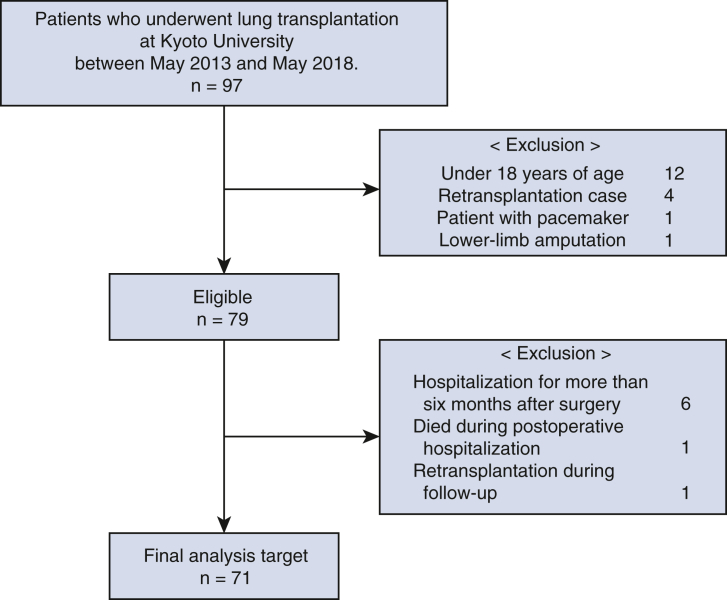
Flow chart of the study participants.

A total of 97 patients underwent lung transplantation at Kyoto University between May 2013 and May 2018, and 79 patients were enrolled in the study. After exclusions, 71 patients (39 males) were included in the analysis (Figure E1).

### Baseline Characteristics

Table 1 presents baseline characteristics, including underlying diseases. Almost one-half of the patients (39 patients) had interstitial lung disease, and 47 patients had undergone brain-dead transplantation. At the time of transplantation, the mean patient age was 48.0 years (IQR, 36.5-56.5 years), and the mean BMI was 19.2 ± 3.6 kg/m^2^. Thirty patients received steroids pretransplantation.

**Table 1:** Baseline characteristics of the study cohort (N = 71)

Characteristic	Value
Age at the time of transplantation, y, median (IQR)	48.0 (36.5-56.5)
Sex, male/female, n	39/32
BMI at the time of transplantation, kg/m^2^, mean ± SD	19.2 ± 3.6
Underlying lung disease diagnoses, n	
Interstitial lung disease	39
Pulmonary complication after hematopoietic stem cell transplantation	9
Pulmonary hypertension	6
Lymphangioleiomyomatosis	6
Chronic obstructive pulmonary disease	4
Bronchiectasis	3
Cartagena syndrome	1
Pulmonary hemosiderosis	1
Cystic fibrosis	1
Castleman's disease	1
Surgical procedures, n	
Bilateral living lung transplantation	24
Brain-dead single lung transplantation	29
Brain-dead bilateral lung transplantation	18
Use of steroids preoperatively, yes/no, n	30/41

*IQR*, Interquartile range; *BMI*, body mass index.

### Changes in Body Composition and Potentially Relevant Data

Table 2 shows the changes in body composition after lung transplantation. With respect to changes in body composition up to 2 years post-transplantation, BMI, FFMI, and FMI changed significantly, with the highest values seen at 1-year post-transplantation. BMI and FFMI increased significantly at 6 months, 1 year, and 2 years after discharge (*P* < .001), with no significant change after 1 year post-transplantation. In contrast, FMI increased significantly at 1 year (*P* < .01) and 2 years (*P* < .001) after discharge, with no significant change after 1 year post-transplantation.

**Table 2:** Changes in body composition and potentially relevant data after lung transplantation

Parameter	At discharge	6 mo	1 y	2 y
Patients who underwent body composition measurement, n	69	65	66	58
Patients who died after discharge, n		0	2	6
Sex, male/female, n	39/30	35/30	35/31	28/30
BMI, overall, mean ± SD	17.8 ± 3.2	19.0 ± 3.3	19.6 ± 3.5	19.2 ± 3.5
Males	18.9 ± 3.2	20.1 ± 3.4	20.8 ± 3.5	20.4 ± 3.2
Females	16.3 ± 2.6	17.7 ± 2.7	18.2 ± 2.9	18.1 ± 3.4
FFMI, overall, mean ± SD	13.8 ± 2.1	14.6 ± 2.1	14.8 ± 2.1	14.6 ± 2.1
Males	15.0 ± 1.8	15.7 ± 1.9	16.1 ± 1.7	16.0 ± 1.9
Females	12.3 ± 1.4	13.3 ± 1.5	13.3 ± 1.4	13.4 ± 1.4
FMI overall, mean ± SD	3.4 (2.4-5.6)	4.1 (2.7-5.7)	4.6 (3.0-6.6)	3.7 (2.7-6.6)
Males	3.4 (2.1-5.3)	4.6 (2.3-5.8)	5.0 (2.3-6.6)	4.3 (2.4-6.6)
Females	3.4 (2.8-5.7)	3.8 (2.7-4.8)	4.1 (3.0-6.3)	3.5 (2.8-6.6)
Nutritional intake				
Energy				
kcal, mean ± SD	2016.3 ± 437.5	1891.0 ± 404.6	1821.5 ± 426.5	1867.1 ± 428.6
kcal/kg IBW, mean ± SD	34.0 ± 6.4	32.3 ± 6.6	30.8 ± 6.2	31.7 ± 6.0
Protein				
g, mean ± SD	75.0 ± 15.6	69.9 ± 16.1	67.7 ± 16.9	68.5 ± 16.0
g/kg IBW, median, (IQR)	1.3 (1.1-1.4)	1.2 (1.0-1.4)	1.1 (1.0-1.3)	1.1 (1.0-1.3)
Missing data	0	3	(5)	(2)
PAL, median (IQR) (missing data)	--- (69)	1.4 (1.3-1.6) (3)	1.4 (1.3-1.5) (5)	1.5 (1.3-1.6) (5)
Steroid medication dose, mg/kg, median (IQR)	0.405 (0.370-0.436)	0.359 (0.303-0.407)	0.149 (0.117-0.198)	0.101 (0.090-0.127)
Steroid pulse therapy after discharge, yes/no, n	---	3/62	6/60	6/52
Insulin administration at discharge, yes/no, n	11/60	---	---	---

Classification of body composition. BMI was considered low if ≤ 18.4 kg/m^2^, standard if 18.5-24.9 kg/m^2^, and high if ≥ 25.0 kg/m^2^. FFMI was considered low if ≤ 16.6 kg/m^2^ in men and ≤14.5 kg/m^2^ in women; standard if 16.7-19.7 kg/m^2^ in men and 14.6-16.6 kg/m^2^ in women; and high if ≥ 19.8 kg/m^2^ in men and ≥16.7 kg/m^2^ in women. BFMI was considered low if ≤ 1.7 kg/m^2^ in men and ≤3.8 kg/m^2^ in women; standard if 1.8-5.1 kg/m^2^ in men and 3.9-8.2 kg/m^2^ in women; and high if ≥ 5.2 kg/m^2^ in men and ≥8.3 kg/m^2^ in women. *BMI*, Body mass index; *FFMI*, fat-free mass index; *FMI*, fat mass index; *IBW*, ideal body weight; *IQR*, interquartile range; *PAL*, physical activity level.

**Figure 1:**
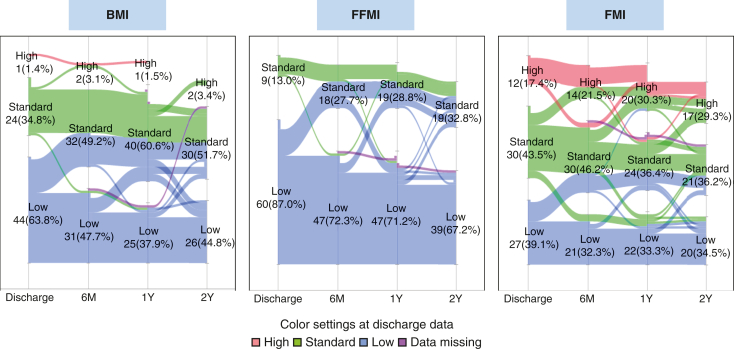
Distribution of low, standard, and high values for each body component and their evolution.

The distributions and transitions of each classification (low, standard, and high) for each body component are shown in Figure 1. Patients with a low BMI were predominant at discharge, but after 6 months, more patients moved above the standard (standard or high) classification. After 1 year, patients with a standard classification were the most common.

The FFMI was low in most patients at discharge, and although some patients reached the standard, 71.2% of patients (47 patients) were still low. Moreover, none of the patients had a high FFMI throughout the study period. In contrast, 17.4% of patients already had high FMI at discharge, and 30.3% had high FMI at 1-year post-transplantation. Nutritional intake was significantly lower at 1 year than at discharge for both total energy (kcal, kcal/kg IBW; *P* < .05) and protein (*P* < .01). PAL was increased significantly (*P* = .041), and steroid medication doses were decreased (*P* < .001), which were managed under the standard dosing protocol at our institution.

### Mortality

The median observation time was 2300 days (IQR, 2027-2773 days), and the median overall survival was 2124 days (IQR, 1397-2576 days), with 15 patients dying during the entire observation period. The causes of death were chronic lung allograft dysfunction (CLAD) in 9 patients, infection in 3 patients, malignancy (other than post-transplantation lymphoproliferative disorder [PTLD]) in 2 patients, and PTLD in 1 patient.

#### Univariate analysis

**Figure 2:**
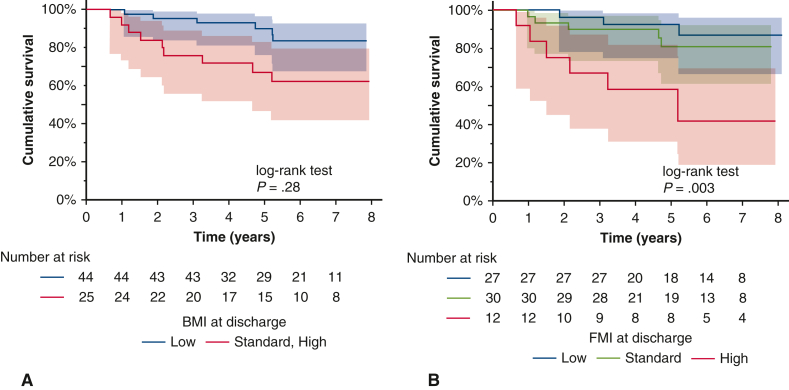
Kaplan‒Meier overall survival curves according to pretransplantation values of body composition. A, Body mass index. B, Fat mass index. The *shaded area* represents the 95% confidence interval.

**Figure 3:**
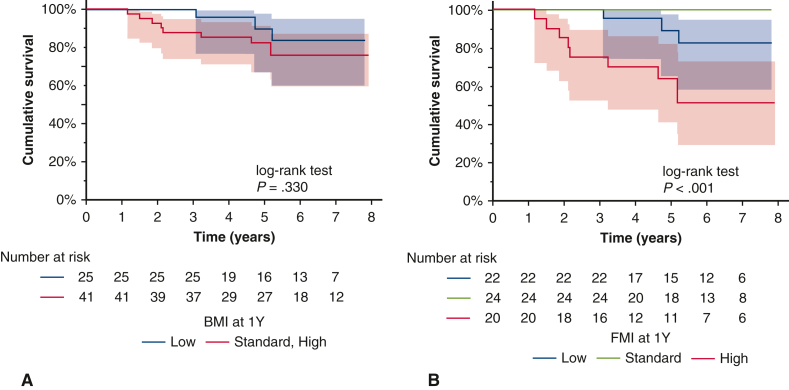
Kaplan‒Meier overall survival curves according to changes in values of body composition at 1 year post-transplantation. A, Body mass index. B, Fat mass index. The *shaded area* represents the 95% confidence interval.

Table 3 shows the associations among body components, potentially relevant covariates, and the number of post-transplantation survival days. We performed the analyses for each body component by dividing them into 3 categories—low, standard, and high—at discharge. Similar analyses were performed at 1 year post-transplantation. Although the BMI is the sum of the FFMI and FMI, we analyzed all 3 because of the numerous reports on the association between BMI and prognosis after lung transplantation. The Kaplan-Meier survival curves are shown in Figures 2 and 3. BMI was divided into low and standard/high groups because only 1 patient had a high BMI at discharge and at 1 year postdischarge.

**Table 3:** Univariate analysis of factors influencing survival after surgery, log-rank test

Variable	Patients, n	Deaths, n	mOS	*P* value
Age				
<40 y	39	4	2181	.019
>50 y	32	11	1903	
Sex				
Male	39	13	2026	.006
Female	32	2	2143	
Underlying lung disease diagnoses				
Interstitial lung disease	39	11	1894	.094
Others	32	4	2208	
BMI at discharge				
Low	44	6	2148	.028
Standard/high	25	9	2026	
FFMI at discharge				
Low	60	13	2118	.885
Standard	9	2	2263	
FMI at discharge				
Low	27	3	2146	.003
Standard	30	5	2144	
High	12	7	1898	
BMI at 1 y				
Low	25	3	2160	.330
Standard/high	41	9	2124	
FFMI at 1 y				
Low	47	7	2139	.316
Standard	19	5	2237	
FMI at 1 y				
Low	22	3	2196	<.001
Standard	24	0	2178	
High	20	9	1796	
Change in FMI from discharge to 1 y				
Low, standard → low, standard	43	3	2206	.003
Low, standard → high	11	4	1428	
Steroid medication doses at 1 y				
Above median	34	6	2171	.837
Below median	32	6	1903	
Steroid pulse therapy during hospitalization				
Yes	32	9	2304	.372
No	39	6	1524	
Steroid pulse therapy from discharge to 1 y				
Yes	6	3	1904	.021
No	60	9	2148	
Steroid pulse therapy up to 1 y postoperatively				
Yes	33	8	2371	.467
No	33	4	1428	
Insulin administration at discharge				
Yes	11	4	1901	.175
No	60	11	2135	

*mOS*, Median overall survival; *BMI*, body mass index; *FFMI*, fat-free mass index; *FMI*, fat mass index.

**Figure E2:**
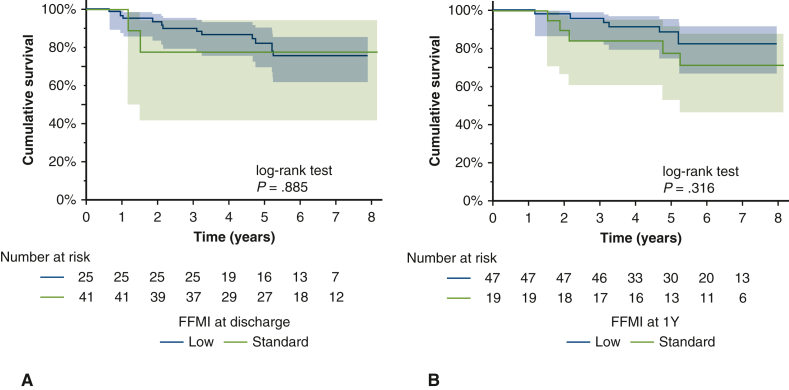
Kaplan-Meier overall survival curves according to pretransplantation or changes in 1-year post-transplantation of fat mass index. The *shaded area* represents the 95% confidence interval.

Among the potentially relevant covariates, age >50 years (*P* = .019), male sex (*P* = .006), and receipt of steroid pulse therapy between discharge and 1 year (*P* = .021) had significantly lower survival rates. In terms of body composition, the standard and high BMI groups at discharge had significantly lower survival rates compared to the low BMI group (*P* = .028), but there was no significant difference in BMI at 1 year post-transplantation. FMI was significantly associated with survival both at discharge (*P* = .003) and at 1 year (*P* < .001) but more significantly at 1 year post-transplantation. Comparisons among the 3 groups revealed significantly lower survival rates in the high group compared to the low and standard groups (at discharge: low vs high, *P* = .002; standard vs high, *P* = .011) (1 year: low vs high, *P* = .021; standard vs high, *P* < .001). There was no significant difference between the low and standard groups, but the low group tended to have lower survival rates compared to the standard group (*P* = .064). None of the patients with standard FMI at 1 year died. FFMI was not significantly associated with survival (Table 3, Figure E2).

**Figure E3:**
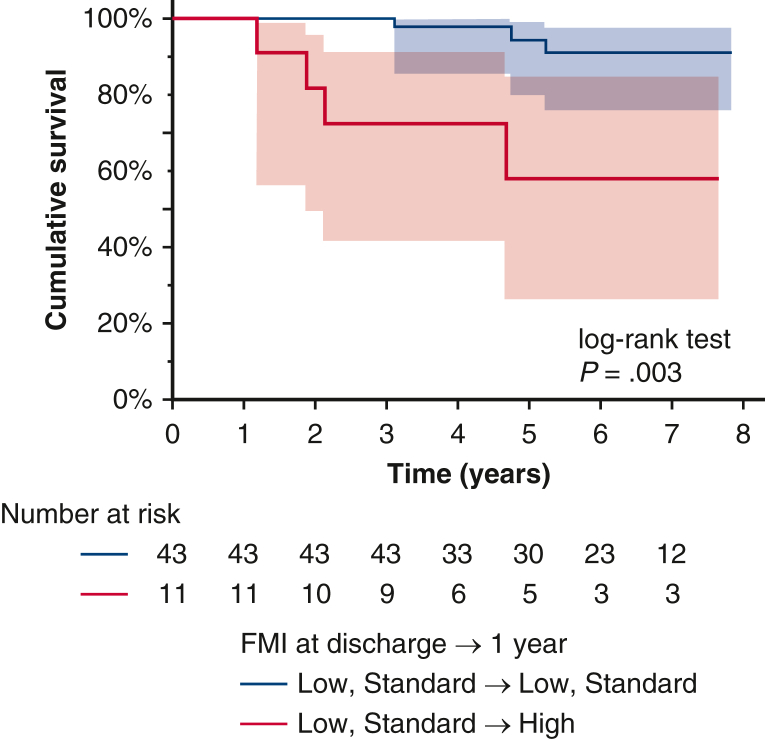
Kaplan-Meier overall survival curves according to changes in group of FMI at discharge to 1-year post-transplantation. The *shaded area* represents the 95% confidence interval.

To verify whether the discharge FMI influences the association between the 1-year post-transplantation FMI and patient prognosis, we excluded the patients with high FMI at discharge. After doing so, the patients who acquired a high FMI at 1 year post-transplantation had a poorer prognosis (Figure E3).

#### Multivariate analysis

Multivariate analysis performed using 2 models for FMI revealed significant differences from the univariate analysis. First, the Cox regression analysis used FMI at discharge and age, sex, diagnosis, and steroid pulse therapy as covariates, with post-transplantation survival days as the dependent variable (Table 4, Model 1). However, no significant variables were obtained. Then Cox regression analysis was performed using 1-year post-transplantation FMI and age, sex, diagnosis, and steroid pulse therapy as covariates, with post-transplantation survival days as the dependent variable (Table 4, Model 2). The results identified FMI at 1-year post-transplantation an independent factor, and the group with a high FMI at 1-year post-transplantation had significantly lower survival (hazard ratio, 5.370; 95% confidence interval, 1.396-20.660; *P* = .014). The causes of death in patients with a high FMI at 1-year post-transplantation were CLAD in 6 patients, infection in 1 patient, and malignancy (other than PTLD) in 2 patients, whereas the cause of death in patients with a low FMI at 1 year post-transplantation was CLAD in 3 patients.

**Table 4:** Predictors of mortality in patients who underwent lung transplantation

Variable	N	β	HR	95% CI	*P* value
Model 1	69				
Age		0.054	1.055	0.993-1.122	.083
Sex					.187
Male	39	1.067	2.908	0.596-14.178	
Female	30	-	1.000	Reference	
Underlying lung disease diagnoses					.675
Interstitial lung disease	38	0.255	1.290	0.392-4.249	
Others	31	-	1.000	Reference	
Steroid pulse therapy during hospitalization					.379
Absence	38	-	1.000	Reference	
Presence	31	0.519	1.680	0.529-5.335	
FMI at discharge					.113
Low/standard	57	-	1.000	Reference	
High	12	0.909	2.482	0.806-7.641	
Model 2	66				
Age		0.089	1.093	0.996-1.201	.061
Sex					.561
Male	35	0.468	1.598	0.329-7.747	
Female	31	-	-	Reference	
Underlying lung disease diagnoses					.365
Interstitial lung disease	37	0.719	2.053	0.433-9.724	
Others	29	-	-	Reference	
Steroid pulse therapy from discharge to 1 y					.288
Yes	33	-	-	Reference	
No	33	0.677	1.968	0.564-6.862	
FMI at 1 y					.014
Low/standard	46	-	-	Reference	
High	20	1.681	5.370	1.396-20.660	

*HR*, Hazard ratio; *CI*, confidence interval; *FMI*, fat mass index.

### Factors Related to Changes in FMI

The 54 patients with low and standard FMI at discharge were studied to determine the influence of the rate of change in the FMI from discharge to 1 year post-transplantation. The associations of the rate of change in FMI with age, sex, underlying lung disease (interstitial lung disease or other), surgical procedure (brain-dead transplant or living donor), nutritional intake at 1 year post-transplantation (energy, kcal and kcal/kg IBW; protein, g and g/kg IBW), PAL, steroid dose at 1 year post-transplantation (mg/kg), presence or absence of steroid pulse therapy from discharge to 1 year post-transplantation, and presence or absence of insulin administration at discharge were subjected to univariate analysis by the Pearson product-moment correlation coefficient, Spearman rank correlation coefficient, *t* test, and Mann-Whitney *U* test, from which factors with *P* < .2 were extracted for multivariate analysis. The extracted factors included age (*P* = .185), sex (*P* = .106), PAL (*P* = .019), steroid dose at 1 year (*P* = .198), and the presence or absence of steroid pulse therapy from discharge to 1 year (*P* = .097). Among these variables, age, sex, PAL, and the presence or absence of steroid pulse therapy were used as independent variables to investigate their influence on the rate of change in the FMI using multiple regression analysis. The results revealed that, although not significantly, PAL (*P* = .052) and the presence or absence of steroid pulse therapy from discharge to 1 year post-transplantation (*P* = .098) tended to be associated with the rate of change in FMI (*R*^2^ = 0.207; *P* = .033, analysis of variance) (Table E1).

## Discussion

In this study, we found a significant time course trend in body composition after lung transplantation. All body composition measures, including BMI, FFMI, and FMI, increased significantly until 2 years post-transplantation and increased the most at 1 year post-transplantation. In addition, a post-transplantation increase in the FMI at 1 year after transplantation may be associated with a poor prognosis.

Our results are consistent with those of a previous report on resting energy expenditure, which showed significant decreases for up to 1 year post-transplantation with no change thereafter.^15^ Surprisingly, even 2 years after surgery, only 32.8% of the patients in the present study reached the FFMI standard, which is lower than that reported in Europe.^16^^,^^19^ Possible reasons for this finding include the smaller body size of Asians and their extremely low FFMI before surgery^6^ and its decrease during the peritransplantation period.^23^ In contrast, the FMI was above the standard for many patients at the time of discharge, and subsequently the number of patients with high levels increased. This prolonged imbalance in body composition also may be influenced by post-transplantation steroid medication, which is known to cause muscle wasting, increased body fat, and obesity.^18^^,^^24^

We highlighted the importance of post-transplantation changes in body composition rather than changes in body weight. Although recent trends in pretransplantation nutrition have focused on skeletal muscle mass and quality,^3^, ^4^, ^5^, ^6^, ^7^ our results emphasize the importance of increasing FMI post-transplantation. In addition to weight maintenance and increasing muscle mass, maintaining or “optimizing” the FMI should be considered desirable during the post-transplantation period.

To develop the optimal nutritional intervention protocol after transplantation, we performed additional analysis of related clinical factors associated with an increase in the post-transplantation FMI. Our analyses show that both PAL and the presence or absence of steroid pulses tended to affect changes in the FMI (Table E1). Although PAL was measured by questionnaire and was not precise, the reported PAL after transplantation was significantly associated with changes in the FMI. PAL may be affected by various factors, including medical condition, physical performance, and environmental conditions, including psychological conditions, and it is likely that optimizing PAL using multimodal interventions is possible. However, we did not find a significant association between nutritional intake assessed by the questionnaire and increased FMI. Although a similar trend was reported in 2011 when only body weight was examined,^25^ it can be postulated that dietary intake assessment after discharge may be less accurate than direct evaluation by a nutritionist during hospitalization.

The presence or absence of steroid pulse therapy tended to be associated with the rate of increase in FMI; however, this association was not statistically significant. Patients who require steroid pulse therapy may experience increased FMI from the effects of the steroid itself, and the reason for using steroid pulse therapy is associated with poor prognosis. Since the present study was a retrospective cohort study, we could not investigate the causal relationship between these treatments and patient outcomes. Further investigation is needed. Nonetheless, based on our observations, we speculate that both a higher FMI at discharge and an increased FMI at 1 year post-transplantation are associated with poor prognosis.

Considering the biological relationship between FM and poor prognosis, an association between leptin levels and the levels of other adipocytokines should be noted. Previous studies have identified pretransplantation obesity as a risk factor for primary graft dysfunction and other complications.^3^^,^^26^^,^^27^ Moreover, inflammation and edema after transplantation may be affected by post-transplantation obesity^28^ and may lead to a poor prognosis.^29^ These circumstances may contribute to the association of the FM trajectory or other body composition parameters with clinical outcomes after lung transplantation.

In this study, the importance of the FFMI, even at discharge and during observation, was unclear. Although previous reports have shown that skeletal muscle mass, including exercise capacity, is associated with better outcomes,^6^ this finding is still consistent with our previous reports demonstrating that changes in skeletal muscle quality, in addition to pretransplantation conditions, are associated with prognosis.^7^^,^^23^ Increasing FM may induce a deterioration in muscle quality, which is related to muscle adiposity.

Given the foregoing, assessing body composition, examining lifestyle factors, and providing appropriate guidance for performing physical activities after lung transplantation are advisable. The BIA method can help provide this multimodal guidance. The method is noninvasive, and the results can be checked immediately after measurement, enabling regular and timely nutritional management and guidance. Recent reports have shown that a comprehensive program of nutrition and rehabilitation can reduce weight and body fat gain and increase muscle mass after lung transplantation.^30^ On the basis of these findings, future interventions and prospective studies using a team-based approach are recommended. Moreover, given that the FMI did not increase after the first post-transplantation year in this study, nutritional guidance during the first year after surgery is particularly important. During this period, more careful and proactive efforts should be made.

The present study has several limitations. First, it was a single-center study with only a few subjects. Future research should explore the long-term effects of team-based interventions on post-transplantation outcomes. It would be valuable to investigate whether enhancing current nutritional guidance during the first post-transplantation year can improve patient outcomes further. Additionally, multicenter studies with larger sample sizes could provide more generalizable results and potentially uncover additional factors influencing post-transplantation recovery. Second, as mentioned previously, we did not directly measure PAL or nutritional intake after discharge. Recent advancements in wearable devices will contribute to research in the field.

In conclusion, after lung transplantation, patients had increased body weight, FFMI, and FMI, but the FFMI remained low in many patients at 1 year post-transplantation. In contrast, the FMI increased above the reference values in an increasing number of patients, and a high FMI at 1 year post-transplantation was associated with a poor prognosis. Therefore, avoiding excess body fat during long-term management following lung transplantation is considered appropriate.

## Conflict of Interest Statement

Dr Sato reports a grant from Nippon Boehringer Ingelheim and grants from Philips-Respironics, Fukuda Denshi, Fukuda Lifetec Keiji, and ResMed with no relevance to the work described herein. All other authors reported no conflicts of interest.

The *Journal* policy requires editors and reviewers to disclose conflicts of interest and to decline handling or reviewing manuscripts for which they may have a conflict of interest. The editors and reviewers of this article have no conflicts of interest.

## Figures and Tables

**Table E1:** Multiple linear regression models of the rate of change in the FMI from discharge to 1 y post-transplantation in lung transplant recipients

Variable	β	*P* value
Age	−0.01	.972
Male sex	0.08	.582
PAL, per 0.1 increase	−0.28	.052
Presence or absence of steroid pulse therapy from discharge to 1-y post-transplantation	0.26	.098

Only patients with low and standard FMI at discharge were analyzed. β represents the standardized regression coefficient. *R*^2^ = 0.207; *P* = .033, analysis of variance. *PAL*, Physical activity level.

## Online Data Supplement Impact of Excessive Body Fat Mass on Mortality After Lung Transplantation

### Food Frequency Questionnaire (FFQ)

Nutritional intake and PAL data were obtained using an FFQ (FFQg version 2.0, Excel Eiyoukun, Kenpakusha), which was administered during nutritional guidance at periodic medical checkups at 6 months, 1 year, and 2 years post-transplantation.^E1^ The FFQ is a questionnaire method for calculating daily nutrient intake based on the average weekly frequency and per-serving intake of each food group 1 to 2 months before the survey date and on the load average nutrient values by food group. From the data thus obtained, the energy and protein contents were extracted and used for analysis.^E1^

PAL (assessed by FFQg version 2.0) were calculated from the time spent in each of the following 6 categories of daily activities: “sleeping,” “lying down or relaxing in a sitting position,” “activities that can be done sitting,” “activities performed standing,” “exercise or work that can be sustained for a long time,” and “exercise or work that requires frequent breaks.” The estimated energy requirement is determined by multiplying the basal metabolic rate calculated from the basal metabolic reference value by PAL, which is classified into 3 levels—low (I), 1.40 to 1.60; standard (II), 1.60 to 1.90; and high (III), 1.90 to 2.20—in the Dietary Reference Intakes for Japanese.^E2^

### Results: Association between Changes in the FMI and Survival

We examined the associations between changes in the FMI from discharge to 1 year post-transplantation and survival. However, because there were 3 FMI groups (low, standard, and high), and because neither low nor high FMI is desirable, analyzing the change in the FMI as a continuous variable was not appropriate. We attempted to examine changes in the FMI by dividing the groups according to how they changed, but this was difficult with 3 groups. Therefore, 54 patients, excluding those with high FMI at discharge, were divided into 2 groups for analysis: those whose FMI changed from low or standard to high and those whose FMI remained low or standard. The results reveal that the participants with low or standard FMI who transitioned to high FMI (11 patients; median overall survival, 1428 days) had a poorer prognosis (*P* = .003) compared to those who maintained a low or standard FMI (43 patients; median overall survival, 2206 days) (Figure E2). The FMI change rate was 105.2% (IQR, 67.2%∼131.7%) for the group that changed from a low or standard to a high FMI and 10.8% (IQR, −5.9%∼46.9%) for the group that maintained a low or standard FMI.
